# Intra-Individual Variability of Vancomycin Trough Concentrations Before and After Implementation of a Standardized Operating Procedure in Orthopedic Inpatients

**DOI:** 10.3390/antibiotics15050517

**Published:** 2026-05-20

**Authors:** Moritz Diers, Laura Isabell Werneburg, Alexander Zeh, Natalia Gutteck, Karl-Stefan Delank, Felix Werneburg

**Affiliations:** 1 Department of Orthopedic and Trauma Surgery, Martin Luther University Halle-Wittenberg, Ernst-Grube-Str. 40, 06120 Halle, Germany; 2 Department of Prosthodontics, Martin Luther University Halle-Wittenberg, Magdeburger Str. 16, 06108 Halle, Germany

**Keywords:** vancomycin, therapeutic drug monitoring, intra-individual variability, orthopedic infections, antibiotic stewardship, standard operating procedure

## Abstract

**Background:** Standardized operating procedures (SOPs) for intravenous vancomycin therapy have been shown to improve population-level trough target attainment and to reduce nephrotoxicity in orthopedic inpatients. However, mean target attainment on a population level does not capture how stably an individual patient remains within the therapeutic window. Intra-individual variability of vancomycin trough concentrations has remained underreported as a patient-level quality indicator in the orthopedic stewardship literature, despite its direct clinical relevance, as alternating sub- and supratherapeutic phases compromise both efficacy and safety independently of the mean exposure. **Methods:** We conducted a secondary analysis of the prospectively and retrospectively collected data of the Halle Vancomycin SOP cohort. Pre-SOP (n = 58) and post-SOP (n = 23) patient cohorts were compared with respect to patient-level variability metrics, including the coefficient of variation (CV%), swing index, mean absolute successive difference (MSSD), range of trough values, zone-transition frequencies, and the proportion of “stable” patients defined as CV% below 20%. First-order Markov transition matrices were computed to characterize the directionality of trough movements between subtherapeutic, target, and supratherapeutic zones. The primary analysis was restricted to patients with at least three documented trough measurements. **Results:** The median CV% decreased from 43.5% (IQR 33.5–51.5) pre-SOP to 32.5% (IQR 21.9–38.6) post-SOP (Mann–Whitney U, *p* = 0.011). The swing index decreased from 1.09 to 0.75 (*p* = 0.002), and the median range of individual trough concentrations shrank from 19.1 mg/L to 13.2 mg/L (*p* = 0.029). The absolute number of zone transitions per patient did not differ significantly between cohorts, but their directionality differed substantially: target-zone persistence increased from 37.8% to 57.6%. Across all 403 measurements, subtherapeutic values declined from 38.5% to 26.6%, while target-zone measurements rose from 28.5% to 44.7%. In the post-SOP cohort, longer therapy duration was associated with lower CV% (Spearman ρ = −0.52, *p* = 0.032). **Conclusions:** In addition to improvements in population-level target attainment, implementation of the SOP was associated with stabilization of the individual exposure profile of orthopedic inpatients receiving intravenous vancomycin. Intra-individual variability was lower in the post-SOP cohort, and transitions between zones were more often oriented toward the target range. These findings, derived from a single-centre secondary analysis with a small post-SOP cohort, support patient-level variability metrics as a complementary quality indicator in protocolized vancomycin management and warrant prospective multicentre validation.

## 1. Introduction

Intravenous vancomycin remains a cornerstone of antimicrobial treatment for serious Gram-positive infections in orthopedic surgery, particularly in the management of periprosthetic joint infections (PJIs), osteomyelitis, and implant-associated complications [1,2]. These clinical scenarios typically require prolonged therapy in patients with substantial comorbidity, and the therapeutic window of vancomycin is narrow. Achieving efficacy while avoiding vancomycin-associated acute kidney injury (VA-AKI) therefore requires disciplined therapeutic drug monitoring (TDM) and dose individualization [3].

Contemporary consensus guidelines by the American Society of Health-System Pharmacists, the Infectious Diseases Society of America, the Pediatric Infectious Diseases Society and the Society of Infectious Diseases Pharmacists recommend an area-under-the-concentration-time-curve to minimum inhibitory concentration ratio (AUC/MIC) of 400–600 mg·h/L as the primary pharmacodynamic target for serious methicillin-resistant *Staphylococcus aureus* (MRSA) infections [4]. In real-world orthopedic practice, however, trough-based monitoring (target 15–20 mg/L) remains the dominant implementation strategy because of limited availability of Bayesian software and clinical pharmacy resources in surgical wards [5,6]. Although meta-analytic data favour AUC-guided monitoring with respect to nephrotoxicity [7], its widespread implementation outside specialized centres remains limited, and the present analysis therefore concerns the optimization of a still-common real-world trough-based practice rather than a challenge to current AUC-based best practice.

The present analysis is conceived as a direct extension of two previous reports from the Halle Vancomycin SOP program, in which we showed that a ward-embedded standardized dosing and monitoring protocol was associated with a reduction in VA-AKI incidence from 17.2% to 0% [8] and with an improvement in trough target attainment from 28.2% to 41.7% across all measurements, alongside a reduction in time-to-target from a median of four to two measurements [9]. Both analyses, however, operated on a population level and did not address a more subtle but clinically important question: whether implementation of the SOP was associated with greater stability of the individual trajectory of trough concentrations over the course of a patient’s therapy.

Population-level target attainment and individual-level exposure stability are conceptually distinct quality indicators: the former describes the aggregate proportion of measurements within the therapeutic range across a cohort, the latter describes the consistency with which a given patient remains within that range over time. A patient whose mean trough concentration lies at 17 mg/L but whose individual measurements oscillate between 8 mg/L and 30 mg/L would, on a population average, be classified as within the therapeutic range—yet this patient is alternately sub- and supratherapeutically exposed over the course of therapy. Subtherapeutic phases jeopardize clinical efficacy, particularly in biofilm-associated infections with high bacterial burdens; supratherapeutic phases increase the risk of nephrotoxicity [3,10]. A more stable intra-individual exposure is therefore an independent quality indicator that is not captured by mean target attainment.

While population pharmacokinetic literature has extensively characterized inter-individual and model-based variability of vancomycin in adult patients [11,12,13], intra-individual trough variability has remained underreported as a patient-level quality indicator in the orthopedic stewardship literature, where population-level target attainment continues to dominate the reported endpoints. In the largest PJI-specific cohort to date, Haglund et al. analyzed 791 trough measurements from 108 patients and reported that 58.2% lay within the 15–20 mg/L target range, but did not characterize the stability of individual patients across their therapy [14]. A similar pattern is observed in institutional guideline evaluations in other settings [6]. This leaves a systematic blind spot between well-documented population-level precision and the clinically decisive exposure trajectory of each patient.

We therefore conducted a secondary analysis of the Halle Vancomycin SOP cohort with the specific aim of characterizing intra-individual trough variability before and after SOP implementation. We hypothesized that implementation of the SOP would be associated with lower intra-individual variability, as measured by the coefficient of variation in trough concentrations per patient, and that the directionality of zone transitions would shift toward the therapeutic target range under protocolized dosing.

## 2. Results

### 2.1. Cohorts and Analytical Population

A total of 81 patients met the core inclusion criteria (pre-SOP n = 58, post-SOP n = 23). Applying the minimum of three documented trough measurements, 51 pre-SOP and 17 post-SOP patients were included in the primary variability analysis, corresponding to 309 and 94 trough measurements, respectively, for a combined total of 403 measurements across both cohorts. Baseline characteristics of both groups corresponded to those previously reported for the Halle Vancomycin SOP cohort [8,9].

### 2.2. Primary and Secondary Variability Metrics

**Figure 1:**
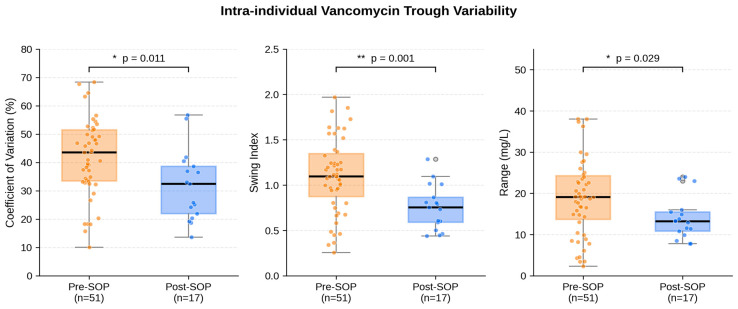
Intra-individual variability of vancomycin trough concentrations. Boxplots display the coefficient of variation (**left**), swing index (**middle**), and absolute range (**right**) per patient for the pre-SOP (orange) and post-SOP (blue) cohorts. Individual data points are overlaid (jittered). Significance indicators: * *p* < 0.05, ** *p* < 0.01.

Patient-level CV% was significantly lower in the post-SOP cohort than in the pre-SOP cohort: median 32.5% (IQR 21.9–38.6) versus 43.5% (IQR 33.5–51.5), Mann–Whitney U *p* = 0.011, with a moderate effect size of r_rb = −0.42. The swing index showed an even stronger reduction, from a median of 1.09 (IQR 0.87–1.35) to 0.75 (IQR 0.59–0.86), *p* = 0.002, r_rb = −0.52. The median absolute range of individual trough concentrations decreased from 19.1 mg/L (IQR 13.7–24.3) to 13.2 mg/L (IQR 10.8–15.5), *p* = 0.029, r_rb = −0.36. The MSSD and the absolute number of zone transitions per patient did not differ significantly between cohorts. The reductions in CV%, swing index, and range showed moderate to medium–large effect sizes (rank-biserial correlation −0.36 to −0.52). The proportion of patients meeting the exploratory stability criterion (CV% < 20%) was numerically higher in the post-SOP cohort (17.6% versus 9.8%) but did not reach statistical significance, most plausibly reflecting limited power in the small post-SOP subgroup rather than absence of an effect. Table 1 summarizes the primary and secondary variability outcomes; Figure 1 provides a graphical overview of the three most informative metrics.

### 2.3. Trough Trajectories and Zone Distribution

**Figure 2:**
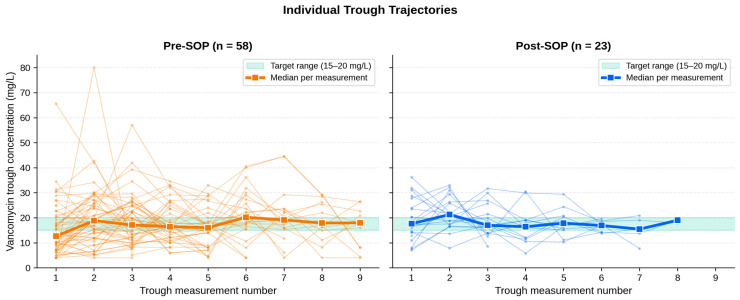
Individual trough trajectories. Spaghetti plots of individual trough measurements across the therapy course, stratified by cohort. Thin lines represent single-patient trajectories; bold lines represent the cohort median at each measurement number. The green band delineates the 15–20 mg/L target range. The narrower clustering of post-SOP trajectories around the target range is visually apparent, both in individual curves and in the cohort-level median.

**Figure 3:**
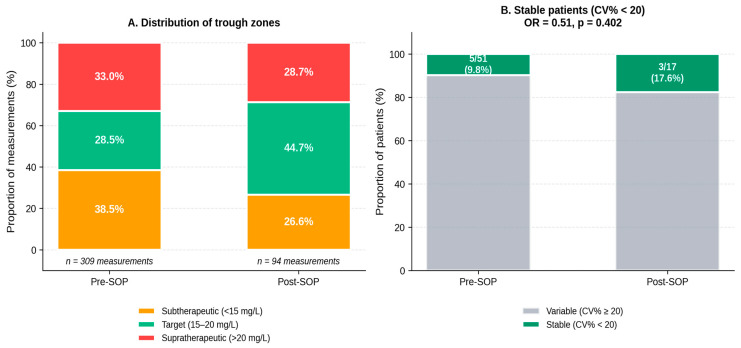
Zone distribution and stability classification. (**A**) Distribution of all documented trough measurements across the three zones. (**B**) Proportion of “stable” patients (defined as CV% < 20% throughout therapy). Owing to small absolute cell counts in the post-SOP cohort (5/51 pre-SOP versus 3/17 post-SOP), this comparison is underpowered and the numerical difference should not be over-interpreted.

Visualization of individual trough trajectories (Figure 2) illustrates the narrower clustering of post-SOP measurements around the 15–20 mg/L target band, both in individual patient curves and in the median per measurement number. Across all 403 measurements, the distribution shifted substantially toward the target zone: subtherapeutic measurements decreased from 38.5% (pre-SOP) to 26.6% (post-SOP), target-zone measurements increased from 28.5% to 44.7%, and supratherapeutic measurements remained nearly stable (33.0% vs. 28.7%). Figure 3 summarizes this distribution and the corresponding stability classification; owing to the small absolute cell counts underlying the stability classification (5/51 pre-SOP versus 3/17 post-SOP), the numerical difference in the proportion of stable patients should be interpreted with caution.

### 2.4. Zone Transition Dynamics

**Figure 4:**
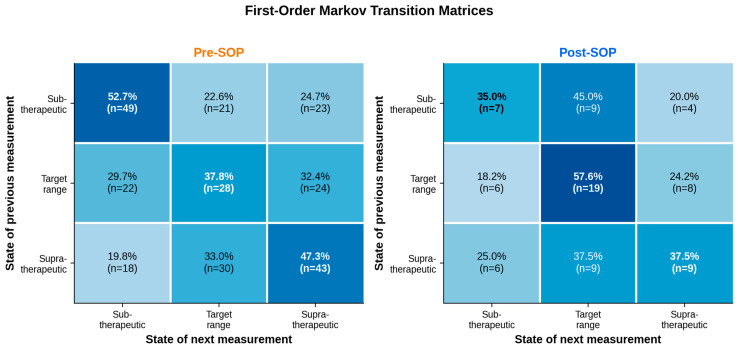
First-order Markov zone-transition matrices. Rows represent the zone of the preceding trough measurement; columns represent the zone of the following measurement. Cells contain the conditional transition probabilities as well as the underlying transition counts. Diagonals (bold) indicate zone persistence. The pooled pre-SOP matrix is derived from n = 258 transitions across 51 patients; the post-SOP matrix is derived from n = 77 transitions across 17 patients. Under the SOP, target-zone persistence increased from 37.8% to 57.6%, and transitions from the subtherapeutic and supratherapeutic zones were more often target-oriented. The matrices are presented for descriptive, hypothesis-generating purposes; no inferential testing of individual transition probabilities was performed.

Although the absolute number of zone transitions did not differ between cohorts, their directionality did. In the pre-SOP cohort, patients starting from a subtherapeutic measurement remained subtherapeutic in the subsequent measurement 52.7% of the time; patients starting from a supratherapeutic measurement remained supratherapeutic 47.3% of the time. In the post-SOP cohort, the dominant pattern was persistence within the target zone (57.6%), combined with target-directed corrective movements from both peripheral zones: 45.0% of subtherapeutic measurements were followed by a target-zone measurement, and 37.5% of supratherapeutic measurements returned to the target zone. The pooled first-order Markov matrices were derived from 258 transitions in the pre-SOP cohort and 77 transitions in the post-SOP cohort, with patients contributing proportionally to their individual trajectory length. As specified in the Materials and Methods, this descriptive analysis was conducted as a hypothesis-generating characterization rather than as inferential testing of transition probabilities. Figure 4 visualizes the full first-order Markov transition matrices.

### 2.5. Exploratory Predictors of Variability in the Post-SOP Cohort

**Figure 5:**
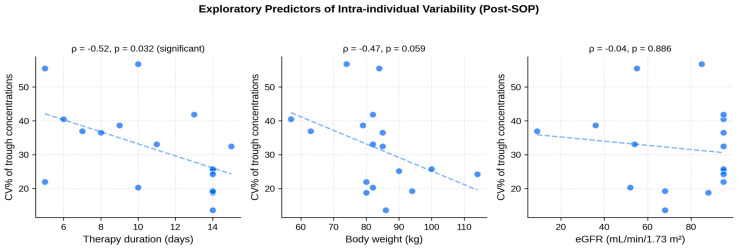
Exploratory predictors of intra-individual variability in the post-SOP cohort. Scatter plots of patient-level CV% against three clinical predictors (n = 17). Dashed lines represent linear regression trends for visualization. Only therapy duration shows a significant negative correlation with CV%.

Within the post-SOP cohort, we explored univariate associations between patient-level CV% and selected clinical covariates. Therapy duration showed a significant negative correlation with CV% (Spearman ρ = −0.52, *p* = 0.032): longer therapies were associated with lower intra-individual variability. Body weight showed a trend in the same direction (ρ = −0.47, *p* = 0.059). Age, eGFR, loading dose in mg/kg, and number of dose adjustments did not correlate significantly with CV%. Figure 5 illustrates the three most informative predictor relationships. These findings are exploratory and should be interpreted with the caveat of the small post-SOP cohort size; no multivariate modeling was performed.

### 2.6. Sensitivity Analyses

When the minimum-measurement criterion was lowered to at least two trough measurements, the cohort sizes increased to 54 pre-SOP and 19 post-SOP patients. The primary effects remained robust: CV% 40.7% versus 32.5% (*p* = 0.011) and swing index 1.09 versus 0.73 (*p* = 0.002). The direction, magnitude and significance of the main findings were independent of the specific minimum-measurement threshold. Within the pre-SOP cohort, stratification by loading-dose receipt did not reveal a significant difference in variability between patients who did and did not receive a weight-based loading dose (CV% 43.6 versus 42.2, *p* = 0.834), arguing against an interpretation that the post-SOP variability reduction was simply a consequence of more consistent loading-dose administration.

## 3. Discussion

### 3.1. Principal Findings

This secondary analysis of the Halle Vancomycin SOP cohort suggests that implementation of protocolized dosing and monitoring was associated not only with improvements in population-level target attainment—as we previously reported [8,9]—but also with greater stability of the individual exposure profile in orthopedic inpatients. Median intra-individual CV% decreased by approximately 11 percentage points, from 43.5% to 32.5%, with a moderate effect size. The swing index and the absolute range of trough concentrations per patient showed concordant reductions. These effects were robust to the choice of the minimum-measurement threshold and were not attributable to the loading-dose component of the SOP alone, as shown by the within-cohort stratification analysis.

A qualitative finding of particular interpretive value is the pattern of zone transitions. The absolute frequency of transitions between subtherapeutic, target, and supratherapeutic zones did not change materially under the SOP. What did change was their directionality: under protocolized care, troughs were more often followed by a return to and persistence within the target zone, whereas pre-SOP patients tended to persist in peripheral zones. Target-zone persistence increased from 37.8% to 57.6%. In other words, under the SOP, oscillation between zones was not eliminated but was more frequently corrected back to the therapeutic target—a dynamic that is invisible to cross-sectional population statistics.

### 3.2. Why Individual Stability Matters Beyond Population Precision

Recent population pharmacokinetic literature has comprehensively characterized inter-individual and model-based variability of vancomycin in adult patients [11,12,13], including substantial heterogeneity in clearance and volume of distribution across published models. The present analysis is complementary rather than redundant to this work: rather than modelling the determinants of inter-individual exposure differences, it characterizes how stably a given patient remains within the therapeutic window over the course of therapy, a quality dimension that has remained underreported in the orthopedic stewardship literature.

The clinical relevance of intra-individual variability has been recognized in pharmacokinetic–pharmacodynamic theory for decades but has received little empirical attention in the orthopedic vancomycin literature. A patient whose trough concentrations oscillate between 8 mg/L and 30 mg/L may, on average, sit within the 15–20 mg/L target range, yet spend substantial intervals in subtherapeutic or supratherapeutic states. Subtherapeutic periods compromise antimicrobial efficacy, particularly in biofilm-associated implant infections where sustained high exposure is required for bacterial clearance [2]. Supratherapeutic periods are closely linked to nephrotoxicity risk, which correlates with cumulative vancomycin exposure and is the primary safety concern of this agent [3,10,15]. Stability of exposure is therefore not a statistical nicety but a genuine clinical quality indicator, complementary to mean target attainment.

Our findings align with—and extend—the observations of Haglund et al. [14], who described the limited reliability of standard weight-based dosing in achieving trough targets in a large PJI cohort but did not analyze patient-level stability. Similar population-level analyses in non-orthopedic settings [6,16] have also refrained from quantifying intra-individual variability. The present analysis therefore contributes a new dimension of evidence by demonstrating that protocolized dosing was associated with reduced per-patient trough variability even in the absence of AUC-guided monitoring, and that this effect is measurable with standard variability metrics derived from the trough data already collected in routine care.

### 3.3. Clinical and Stewardship Implications

For orthopedic departments implementing vancomycin SOPs, our findings provide two practical messages. First, the clinical benefit of protocolized dosing extends beyond population-level target attainment to include stabilization of individual exposure trajectories. This supports the use of patient-level stability as an additional quality indicator in antibiotic stewardship monitoring, alongside conventional target-attainment rates. Second, the directionality of zone transitions offers a diagnostic lens on the protocol’s dose-adjustment matrix: persistent out-of-range states would signal insufficient corrective action, while target-oriented transitions—as observed here—indicate that the adjustment logic is operating as designed. Routine audit of transition patterns could therefore provide actionable feedback for protocol refinement.

The exploratory negative association between therapy duration and CV% in the post-SOP cohort should be interpreted cautiously. It does not imply that longer therapy duration itself reduces variability; alternative explanations include a greater number of opportunities for dose refinement in longer trajectories, as well as a selection of patients with more complex courses for whom systematic dosing attention may have been particularly attentive. The exploratory nature of the analysis and the small post-SOP cohort preclude causal inference.

A natural question is which elements of the SOP contributed most to the observed stabilization. The protocol combines a mandatory weight-based loading dose, renal-function-adjusted maintenance dosing, scheduled TDM with renal-function-dependent timing of the first trough, and a structured dose-adjustment matrix. The within-cohort sensitivity analysis stratified by loading-dose receipt argued against a dominant effect of the loading dose in isolation, suggesting that structured timing of TDM and the dose-adjustment matrix—particularly the latter—are plausible drivers of the observed transition-directionality shift. The present data, however, cannot disentangle the relative contributions of these components, and a controlled evaluation of individual SOP elements remains a question for prospective work.

### 3.4. Limitations

Several limitations temper the interpretation of our findings. First, the present analysis is a single-centre retrospective secondary analysis with a small post-SOP cohort (n = 17 in the primary variability analysis); the corresponding effect estimates should be interpreted as exploratory, and the numerical increase in the stable-patient proportion (9.8% to 17.6%) did not reach statistical significance, most plausibly reflecting limited power rather than absence of an effect. Second, the retrospective pre-post design does not permit causal attribution; temporal confounding by broader quality-improvement developments at the institution—including changes in ward practice, monitoring behaviour, and general stewardship awareness—cannot be excluded, and the observed associations should accordingly not be ascribed to the SOP in isolation. Third, the binary “stable patient” classification using a CV% < 20% threshold reflects a general TDM convention rather than a vancomycin-specific validated cut-off and is therefore presented as an exploratory descriptor only. Fourth, the ≥3-measurement inclusion criterion enriches the analyzed population for patients with longer or more complex therapy courses; findings are therefore most directly applicable to sustained inpatient vancomycin therapy. Fifth, within the pooled first-order Markov matrices, patients with longer trajectories contributed proportionally more transitions; a patient-weighted re-analysis was not performed in the present secondary analysis and represents a methodological refinement appropriate for future prospective work with larger cohorts. Sixth, the exact timing of individual trough measurements was based on administrative documentation in the electronic medical record (ORBIS); minor deviations between documented and true sampling times cannot be excluded [17], and inter-laboratory analytical variability of vancomycin immunoassays has been reported in external quality assurance data [18]. Both factors may contribute to a residual analytical and pre-analytical component of the observed variability and predominantly affect interval-sensitive metrics such as MSSD rather than the dimensionless CV% or swing index. Seventh, surgical sub-type and detailed indication-specific variables were not consistently recorded in the source dataset; combined with the small post-SOP cohort, this precluded meaningful multivariable modelling or stratified subgroup analysis. Eighth, the analysis did not incorporate minimum inhibitory concentrations of the isolated pathogens and therefore relates exclusively to trough variability, not to AUC/MIC variability; integration of AUC-guided analysis remains a desirable next step once Bayesian pharmacokinetic infrastructure becomes routinely available [19,20]. Finally, because several secondary and exploratory comparisons are reported without correction for multiple testing, all secondary findings should be interpreted with corresponding caution.

### 3.5. Future Directions

Three complementary avenues of investigation follow naturally from the present results. First, extending the analysis to a larger and ideally multicentre post-SOP cohort would enable multivariable modelling of variability determinants—including baseline renal function, age, surgical sub-type, and indication-specific covariates—and would clarify whether the observed trends, particularly the association with therapy duration, hold in a broader sample. Second, future prospective studies should explicitly link individual transition trajectories with patient-level safety and efficacy outcomes, such as bacteremia duration, treatment failure, relapse, and the incidence of vancomycin-associated acute kidney injury; aggregate safety and efficacy outcomes for the present cohort have been reported previously [8,9], but a direct intra-patient linkage between exposure trajectory and outcome is beyond the scope of the present secondary analysis and remains an important next step, particularly in the PJI-specific setting where exposure-outcome data remain limited [21]. Third, incorporating Bayesian AUC reconstruction in subsequent analyses would place the stability findings within the pharmacodynamic framework that currently defines best practice for vancomycin therapy [4,19,20]. A multicentre replication of the variability-based quality endpoint across different institutional settings would further test the transferability of the observed SOP-associated stabilization beyond a single academic department.

## 4. Materials and Methods

### 4.1. Study Design

This study is a single-center retrospective pre-post cohort analysis performed at the Department of Orthopedic and Trauma Surgery of the University Hospital Halle (Saale), Germany. It relies exclusively on secondary analysis of the anonymized patient data that formed the basis of our two previous reports [8,9]; no new data were collected. The pre-SOP cohort was retrospectively identified from the institutional pharmacy and TDM databases; the post-SOP cohort was prospectively documented under the protocol implemented in March 2024.

### 4.2. Patient Selection

Inclusion criteria were identical to those used in the two previous reports: adult orthopedic inpatients receiving intravenous vancomycin for at least 72 h as empirical combination therapy (typically ampicillin/sulbactam plus vancomycin) or as targeted monotherapy for microbiologically confirmed infection. Exclusion criteria were single-dose administration, therapy shorter than 72 h, severe baseline renal dysfunction (chronic kidney disease KDIGO stage 4 or higher; eGFR < 30 mL/min/1.73 m^2^), dialysis dependence, concurrent nephrotoxic co-medication (aminoglycosides, NSAIDs), incomplete documentation of trough levels or renal function, and therapy initiated or continued outside the orthopedic service.

For the present variability analysis, patients were further required to have at least three documented trough measurements to ensure statistically valid intra-individual variability estimation. This minimum-measurement criterion is a methodological requirement because the coefficient of variation is unstable when computed from only two data points. Clinically, this criterion enriches the analyzed population for patients with longer or more complex therapy courses; the findings should therefore be regarded as most directly applicable to sustained inpatient vancomycin treatment rather than to the full spectrum of orthopedic vancomycin use. Sensitivity analyses with a reduced minimum of two measurements were performed to assess robustness.

### 4.3. Standardized Operating Procedure

The Halle Vancomycin SOP, implemented in March 2024, has been described in detail elsewhere [8,9]. Its core components are a mandatory weight-based loading dose of 25 mg/kg, renal-function-adjusted maintenance dosing, a predefined target trough range of 15–20 mg/L, and scheduled TDM: the first trough was drawn within one hour before the fourth dose in patients with eGFR ≥ 40 mL/min/1.73 m^2^, before the third dose in patients with eGFR 20–39 mL/min/1.73 m^2^, and 24 h after the loading dose in patients with eGFR < 20 mL/min/1.73 m^2^ or on dialysis. Dose adjustments followed a structured matrix integrating trough level and renal function.

### 4.4. Laboratory Methods and Sampling

Vancomycin trough concentrations were determined using the routine validated assay of the central laboratory of the University Hospital Halle. Sampling times were documented electronically in the institutional medical record system (ORBIS) and used as the administrative reference time of trough measurement. Minor deviations between documented and true sampling times cannot be excluded; mistimed trough sampling has been described as a recognized source of variability in routine therapeutic drug monitoring with potential clinical consequences [17], and inter-laboratory analytical variability of vancomycin immunoassays has been documented in recent external quality assurance data [18]. Both factors may contribute to a residual analytical and pre-analytical component of the observed intra-individual trough variability.

### 4.5. Variability Metrics

For each patient, we computed the following variability metrics from the full sequence of documented trough concentrations. The coefficient of variation (CV%) was defined as the standard deviation of trough values divided by the arithmetic mean, expressed as a percentage, and served as the primary variability measure. It is dimensionless, pharmacologically interpretable, and robust to differences in mean exposure between patients. The swing index was defined as (Cmax − Cmin)/Cmean per patient, sensitive to extremes and clinically intuitive as the peak-to-trough amplitude relative to average exposure. The mean absolute successive difference (MSSD) was computed as the mean of absolute differences between consecutive trough measurements per patient, reflecting short-term dynamics between adjacent time points. A binary stable patient classification was set to 1 if CV% was below 20% across the full therapy. The 20% threshold reflects the conventionally cited boundary for acceptable intra-individual pharmacokinetic variability of narrow-therapeutic-index drugs in therapeutic drug monitoring; this threshold has not been formally validated in a vancomycin-specific context and is therefore used here as a pragmatic exploratory descriptor rather than a validated clinical category. Each trough was classified as subtherapeutic (<15 mg/L), target (15–20 mg/L), or supratherapeutic (>20 mg/L); zone transitions between consecutive measurements were counted per patient, and first-order Markov transition matrices were computed for each cohort, expressing the probability of moving from one zone to another. The Markov transition analysis is descriptive and hypothesis-generating in nature; no inferential testing of individual transition probabilities was applied. Within each cohort, patients with longer therapy trajectories contributed proportionally more transitions to the pooled matrix; a patient-weighted re-analysis was not performed in the present secondary analysis (see Limitations). The total number of zone-to-zone transitions contributing to each cohort matrix was 258 (pre-SOP) and 77 (post-SOP).

### 4.6. Outcome Measures

The primary outcome was the comparison of patient-level CV% between the pre- and post-SOP cohorts. Secondary outcomes were comparisons of swing index, MSSD, trough range, number of zone transitions, and the proportion of stable patients. Exploratory outcomes included univariate associations between CV% and clinical covariates (age, sex, eGFR, body weight, therapy duration, number of dose adjustments) within the post-SOP cohort, and the first-order Markov transition matrices.

### 4.7. Statistical Analysis

The present manuscript constitutes a secondary, exploratory analysis of the Halle Vancomycin SOP cohort; the underlying cohort was originally sized to support the primary endpoints (VA-AKI incidence, population-level target attainment) of the two parent publications [8,9]. Because the variability and zone-transition endpoints examined in the present analysis are new, distinct, and exploratory, no a priori power calculation was performed. Continuous variables are summarized as median with interquartile range (IQR) due to the expected right-skewness of variability measures; normality was assessed using the Shapiro–Wilk test. Between-cohort comparisons of continuous variables used the Mann–Whitney U test with rank-biserial correlation (r_rb) as an effect-size measure. Categorical comparisons used Fisher’s exact test. Exploratory correlations used Spearman’s rho. Zone transition matrices were computed by concatenating all consecutive zone-to-zone transitions across patients within each cohort. All *p*-values are two-sided, with α = 0.05 considered statistically significant. Given the exploratory nature of secondary comparisons, no formal correction for multiple testing was applied; results are reported transparently for reader interpretation and should be regarded as hypothesis-generating rather than confirmatory. Analyses were performed in IBM SPSS Statistics for Windows, Version 27.0 (IBM Corp., Armonk, NY, USA) and in Python 3.12 (Python Software Foundation, Wilmington, DE, USA) using pandas 2.2.3, SciPy 1.14.1, and Matplotlib 3.9.2 for computation of variability metrics and visualization.

### 4.8. Ethical Approval

The use of anonymized data from the Halle Vancomycin SOP cohort was covered by the declaration of no objection issued by the Ethics Committee of the Medical Faculty at Martin Luther University Halle-Wittenberg (reference 2025-129). Because this secondary analysis used exclusively the previously approved anonymized dataset and applied only additional statistical methods without new data collection, no further ethical approval was required.

## 5. Conclusions

In this single-centre secondary analysis with a small post-SOP cohort, implementation of a structured vancomycin SOP in orthopedic inpatients was associated with lower intra-individual trough variability and with a more target-oriented pattern of zone transitions, complementing the population-level improvements in target attainment and safety previously reported for the same cohort [8,9]. While the present findings should be interpreted cautiously given the exploratory design and limited sample size, they support patient-level variability metrics as a complementary quality indicator in protocolized vancomycin management and motivate prospective multicentre validation alongside outcome-linked analyses.

## Figures and Tables

**Table 1:** Primary and secondary variability metrics, pre-SOP versus post-SOP (patients with ≥3 measurements).

Metric	Pre-SOP (n = 51)	Post-SOP (n = 17)	*p*-Value	r_rb
**CV% (median, IQR)**	43.5 (33.5–51.5)	32.5 (21.9–38.6)	0.011	−0.42
**Swing index (median, IQR)**	1.09 (0.87–1.35)	0.75 (0.59–0.86)	0.002	−0.52
**Range in mg/L (median, IQR)**	19.1 (13.7–24.3)	13.2 (10.8–15.5)	0.029	−0.36
**MSSD in mg/L (median, IQR)**	8.1 (4.9–10.3)	5.7 (3.7–10.5)	0.615	−0.08
**Zone transitions (median, IQR)**	2 (2–4)	2 (2–3)	0.638	−0.08
**Mean trough in mg/L**	17.5 (14.5–20.4)	17.9 (16.7–20.6)	0.376	+0.15
**Stable patients (CV% < 20), n (%)**	5/51 (9.8%)	3/17 (17.6%)	0.402 *	—

* Fisher’s exact test. CV% = coefficient of variation; IQR = interquartile range; MSSD = mean absolute successive difference; r_rb = rank-biserial correlation effect size. Negative r_rb indicates lower values in the post-SOP cohort.

## Data Availability

The data presented in this study are available on request from the corresponding author. The data are not publicly available due to privacy and institutional restrictions.
